# Application of a Weighted Absolute Percentage Error-Based Method for Calculating the Aggregate Accuracy of Reported Malaria Surveillance Data

**DOI:** 10.4269/ajtmh.24-0804

**Published:** 2025-04-22

**Authors:** Smita Das, Arantxa Roca-Feltrer, Michael Hainsworth

**Affiliations:** ^1^ PATH Malaria Control and Elimination Partnership (MACEPA), Seattle, Washington;; ^2^ PATH MACEPA, Maputo, Mozambique

## Abstract

Aggregate malaria data reporting accuracy indicates the overall quality of reported malaria surveillance data and is calculated using a routine data quality audit (RDQA) toolkit during a health facility audit. Three example scenarios are presented that highlight the limitations of the aggregate reporting accuracy methods of three malaria RDQA toolkits. A weighted absolute percentage error-based aggregate data reporting accuracy (WADRA) approach was found to resolve these limitations by using the register values as the weighting factor, enabling the detection of low-accuracy facilities that are otherwise considered high-accuracy by the current toolkits. Accordingly, country malaria programs should consider adopting the WADRA method in their RDQAs to maximize the detection of low-accuracy facilities and enhance decisions ranging from the development and implementation of corrective action plans to the prioritization and allocation of resources for data quality improvement efforts.

Malaria surveillance data are the foundation for a wide range of decisions made by country malaria programs that aim to optimize responses by directing resources and appropriate interventions to populations most in need, enhancing program planning and implementation, and supporting advocacy for domestic and international investments.^1^ Therefore, it is essential that high-quality routine data captured in a country’s health information system are reliable, consistent, and actionable. Malaria programs are expected to routinely monitor a set of indicators that measure the quality of reported surveillance data; these core indicators include reporting rate, reporting completeness, timeliness, accuracy, consistency, and validity. Two separate processes are implemented to review these indicators across key malaria data elements: 1) a regular desk review of existing aggregated reported data for most indicators and 2) a routine data quality audit (RDQA) of aggregated reported data with source documents at the service-delivery level for the accuracy indicator. This makes the RDQA a critical activity for identifying and correcting errors, as well as recognizing successful practices and systematic challenges in the reporting process.

Currently, two toolkits are widely used in malaria-endemic countries for conducting malaria RDQAs: the MEASURE Evaluation Malaria Routine Data Quality Assessment Tool and the WHO Data Quality Assurance Toolkit.^2^^,^^3^ Additionally, the PATH Malaria Control and Elimination Partnership (MACEPA) has developed the PATH MACEPA RDQA Toolkit, which is currently being used for malaria RDQAs in select regions of Zambia, Senegal, and the Democratic Republic of Congo.^4^ Table 1 summarizes the key attributes of each toolkit. Although most attributes are similar across the tools, some differences were observed, particularly in the aggregate reporting accuracy method; both the MEASURE Evaluation and the WHO toolkits calculate an average verification factor (VF) based on individual month VFs:

$${VF}_{i}=\frac{\text{Register}\;\text{value}}{\text{Reported}\;\text{value}}\;\text{such}\;\text{that}\;\bar{VF}=\frac{\sum_{i=1}^{n}{VF}_{i}}{n}\;\times 100$$

where *n* is the number of months. The PATH toolkit calculates aggregate reporting accuracy using the following formula:

$$\text{Accuracy}=\;\frac{\text{Minimum}\;\text{sum}\;\text{value}\;\left(\sum_{i=1}^{n}y_{i}\;or\;\sum_{i=1}^{n}{\hat{y}}_{i}\;\right)}{\text{Maximum}\;\text{sum}\;\text{value}\;\left(\sum_{i=1}^{n}y_{i}\;or\;\sum_{i=1}^{n}{\hat{y}}_{i}\;\right)}\;\times 100$$

where *n* represents the number of reporting periods, $y_{i}$ represents the register value, and ${\hat{y}}_{i}$ represents the reported value.

**Table 1:** Summary of toolkits used for routine data quality audits of reported malaria surveillance data

RDQA Tool Attributes	MEASURE Evaluation Malaria Routine Data Quality Assessment Tool	WHO Data Quality Assurance Toolkit	PATH MACEPA RDQA Toolkit
Purpose	Support routine review of malaria data quality at health facilities by district teams	Establish a harmonized approach and standardized language for evaluating and strengthening health facility data quality	Support routine review of data quality at health facilities and districts.
Disease focus	Malaria	Not disease-specific (recommended: maternal health, immunization, HIV, TB, and malaria)	Not disease specific
When should RDQAs be conducted	As part of routine supportive supervision	As part of routine supportive supervision or discrete/cross-sectional assessment	As part of routine supportive supervision or discrete/cross-sectional assessment
Target health level	District; Health facility	District; Health facility	District; Health facility
Method of health facility and/or district selection	Random and purposive selection of up to 24 health facilities	Based on sample size calculation and random sampling	Random and purposive selection
Expected frequency of implementation	Quarterly	Quarterly	As determined by national malaria program
Months of data reviewed	3 months	3 months	As determined by national malaria program
RDQA tool format	Excel-based	Excel-based	Excel-based
RDQA data quality indicators assessed	1. Timeliness	1. Timeliness	1. Reporting rate
	2. Completeness	2. Completeness	2. Timeliness
	3. Indicator accuracy	3. Indicator accuracy	3. Completeness
	4. Cross-checks	4. Consistency over time	4. Reporting period accuracy
	5. Consistency over time	5. System assessment	5. Indicator accuracy
	6. System assessment	–	–
Maximum number of indicators that can be evaluated	Up to 5 indicators	Five indicators (recommended: antenatal care first visit coverage, DTP3/Penta3 coverage, new on ART, TB notification rate, confirmed malaria cases)	As determined by national malaria program
Data reporting accuracy metric for individual unit of time audited	${VF}_{i}=\frac{\text{Register}\;\text{value}}{\text{Reported}\;\text{value}}\;\times 100$	${VF}_{i}=\frac{\text{Register}\;\text{value}}{\text{Reported}\;\text{value}}\;\times 100$	$\text{Accuracy}=\;\frac{\text{Minimum}\;\text{value}\;\left(\text{register}\;or\;\text{reported}\;\text{values}\right)}{\text{Maximum}\;\text{value}\;\left(\text{register}\;or\;\text{reported}\;\text{values}\right)}\;\times 100$
Aggregate data reporting accuracy metric per audit period	$\bar{VF}=\frac{\sum_{i=1}^{n}{VF}_{i}}{n}\;\times 100$ where *n* is the number of months	$\bar{VF}=\frac{\sum_{i=1}^{n}{VF}_{i}}{n}\;\times 100$ where *n* is the number of months	$\text{Accuracy}=\;\frac{\text{Minimum}\;\text{sum}\;\text{value}\;\left(\sum_{i=1}^{n}y_{i}\;or\;\sum_{i=1}^{n}{\hat{y}}_{i}\;\right)}{\text{Maximum}\;\text{sum}\;\text{value}\;\left(\sum_{i=1}^{n}y_{i}\;or\;\sum_{i=1}^{n}{\hat{y}}_{i}\;\right)}\;\times 100$ where *n* is the number of reporting periods, $y_{i}$ represents the register value, and ${\hat{y}}_{i}$ represents the reported value

ART = antiretroviral therapy; DTP3 = diptheria tetanus pertussis vaccine third dose; HIV = human immunodeficiency virus; MACEPA = Malaria Control and Elimination Partnership; Penta3 = pentavalent vaccine third dose; RDQA = routine data quality audit; VF = verification factor; TB = tuberculosis.

Both approaches have advantages; for example, the average verification factor ($\bar{VF}$) indicates the extent of overreporting ($\bar{VF}$ less than 0.90) and underreporting ($\bar{VF}$ greater than 1.10) by a health facility during an audit period (MEASURE Evaluation 2020). The PATH toolkit’s aggregate reporting accuracy method emphasizes how closely the total reported value matches the total register value. It is categorized into one of three reporting accuracy levels: low (<70%), medium (70–85%), and high (>85%). This makes it a simple metric that district (or higher) level staff can promptly interpret to address data quality issues.

However, there are situations in which the aggregate reporting accuracy output from all three toolkits incorrectly classifies health facilities as reporting with high accuracy. This limitation and its impact on data quality improvement efforts are highlighted in Tables 2–4 through three example RDQA scenarios for confirmed malaria cases over a 3-month reporting period.

**Table 2:** Example scenario of confirmed malaria cases during a 3-month period that highlights the limitation with current RDQA toolkits’ approaches to measuring aggregate data reporting accuracy where errors between register and reported values cancel each other out

RDQA Data					
Audit Month	Register Value	Reported Value	Difference (register-reported)	Absolute Difference (register-reported)	VF
Month 1	3	3	0	0	1.00
Month 2	2	7	−5	5	0.29
Month 3	12	7	5	5	1.71
Total	17	17	0	10	–

Aggregate Data Reporting Accuracy Calculations			
Aggregate Data Reporting Accuracy Calculation Method	Aggregate Data Reporting Accuracy	Low/High Aggregate Data Reporting Accuracy	Low/High Prioritization for Data Quality Improvement Activities
MEASURE Evaluation Toolkit ($\bar{VF})$	1.00	High	Low
WHO Toolkit ($\bar{VF})$	1.00	High	Low
PATH Toolkit (%)	100%	High	Low
WADRA (%)	41	Low	High

RDQA = routine data quality audit; VF = verification factor; $\bar{VF}$ = average verification factor; WADRA = weighted absolute percentage error-based aggregate data reporting accuracy.

**Table 3:** Example scenario of confirmed malaria cases during a 3-month period that highlights a limitation with current RDQA toolkits’ approaches to measuring aggregate data reporting accuracy where equal importance is given to health facilities regardless of register values

Health Facility #1						Health Facility #2					
RDQA Data						RDQA Data					
Audit Month	Register Value	Reported Value	Difference (register-reported)	Absolute Difference (register-reported)	VF	Audit Month	Register Value	Reported Value	Difference (register-reported)	Absolute Difference (register-reported)	VF
Month 1	1	1	0	0	1.00	Month 1	115	116	−1	1	0.99
Month 2	1	1	0	0	1.00	Month 2	101	210	−109	109	0.48
Month 3	5	4	1	1	1.25	Month 3	300	170	130	130	1.76
Total	7	6	1	1	–	Total	516	496	20	240	–

Aggregate Data Reporting Accuracy Calculations				Aggregate Data Reporting Accuracy Calculations			
Aggregate Data Reporting Accuracy Calculation Method	Aggregate Data Reporting Accuracy	Low/High Aggregate Data Reporting Accuracy	Low/High Prioritization for Data Quality Improvement Activities	Aggregate Data Reporting Accuracy Calculation Method	Aggregate Data Reporting Accuracy	Low/High Aggregate Data Reporting Accuracy	Low/High Prioritization for Data Quality Improvement Activities
MEASURE Evaluation Toolkit ($\bar{VF})$	1.08	High	Low	MEASURE Evaluation Toolkit ($\bar{VF})$	1.08	High	Low
WHO Toolkit ($\bar{VF})$	1.08	High	Low	WHO Toolkit ($\bar{VF})$	1.08	High	Low
PATH Toolkit (%)	86	High	Low	PATH Toolkit (%)	96	High	Low
WADRA (%)	86	High	Low	WADRA (%)	53	Low	High

RDQA = routine data quality audit; VF = verification factor; $\bar{VF}$ = average verification factor; WADRA = weighted absolute percentage error-based aggregate data reporting accuracy.

**Table 4:** Example scenario of confirmed malaria cases during a 3-month period that highlight a limitation with current RDQA toolkits’ approaches to measuring aggregate data reporting accuracy where small differences in reporting values and extreme verification factor values can significantly impact the average verification factor

Health Facility #3						Health Facility #4					
RDQA Data						RDQA Data					
Audit Month	Register Value	Reported Value	Difference (register-reported)	Absolute Difference (register-reported)	VF	Audit Month	Register Value	Reported Value	Difference (register-reported)	Absolute Difference (register-reported)	VF
Month 1	9	2	7	7	4.50	Month 1	9	3	6	6	3.00
Month 2	1	11	−10	10	0.09	Month 2	1	11	−10	10	0.09
Month 3	1	116	−115	115	0.01	Month 3	1	116	−115	115	0.01
Total	11	129	−118	132	–	Total	11	130	−119	131	–

Aggregate Data Reporting Accuracy Calculations				Aggregate Data Reporting Accuracy Calculations			
Aggregate Data Reporting Accuracy Calculation Method	Aggregate Data Reporting Accuracy	Low/High Aggregate Data Reporting Accuracy	Low/High Prioritization for Data Quality Improvement Activities	Aggregate Data Reporting Accuracy Calculation Method	Aggregate Data Reporting Accuracy	Low/High Aggregate Data Reporting Accuracy	Low/High Prioritization for Data Quality Improvement Activities
MEASURE Evaluation Toolkit ($\bar{VF})$	1.53	Low (underreporting)	High	MEASURE Evaluation Toolkit ($\bar{VF})$	1.03	High	Low
WHO Toolkit ($\bar{VF})$	1.53	Low (underreporting)	High	WHO Toolkit ($\bar{VF})$	1.03	High	Low
PATH Toolkit (%)	0	Low	High	PATH Toolkit (%)	0	Low	High
WADRA (%)	0	Low	High	WADRA (%)	0	Low	High

RDQA = routine data quality audit; VF = verification factor; $\bar{VF}$ = average verification factor; WADRA = weighted absolute percentage error-based aggregate data reporting accuracy.

In the first scenario (Table 2), errors between the register and reported values at a health facility cancel each other when calculating the aggregate reporting accuracy. Although there are differences in the register and reported values in Month 2 and Month 3, all three tools show 100% reporting accuracy, indicating high data quality.

The second scenario (Table 3) shows how the current aggregate reporting accuracy methods assign equal importance to health facilities, regardless of their caseloads. Health Facility #1, which has few confirmed malaria cases and small differences in register and reported values, would receive the same prioritization and resources for corrective actions as Health Facility #2, which has a high number of malaria cases and large differences in register and reported values. When resources are limited, the aggregate accuracy metric should prioritize errors at facilities with higher caseloads (Health Facility #2) to guide the most effective distribution of resources for improving data quality. However, in an elimination setting, it is essential to identify and resolve the issues affecting data quality at Health Facility #1 to ensure that all malaria cases are captured.

The third scenario (Table 4) demonstrates how small differences in reporting values and relatively extreme VFs can have considerable impact on the $\bar{VF}\;$when using the MEASURE Evaluation and WHO toolkits. For the $\bar{VF}$ of Health Facility #3, comparison of the register and reported values show underreporting in Month 1 (VF = 4.5) and significant overreporting in Month 2 (VF = 0.09) and Month 3 (VF = 0.01). Because Month 1 VF is so high, it drives most of the facility’s $\bar{VF}\;$to 1.53, suggesting considerable underreporting. For the $\bar{VF}$ of Health Facility #4, the same register and reported values from the Health Facility #3 $\bar{VF}\;$scenario were used, with the exception of Month 1, during which the reported value increased by one confirmed malaria case. This increase resulted in a high Month 1 VF of 3.00 (high underreporting), and together with the VFs of Months 2 and 3, the $\bar{VF}\;$is 1.03, suggesting that Health Facility #4 is reporting with high accuracy. It is concerning that a difference of one malaria case between both health facilities, along with the associated high VFs in Month 1, can lead to such variable outputs and prioritizations. The PATH toolkit aggregate reporting accuracy for both health facilities is 9%, signifying low performance and triggering the further examination of surveillance practices.

Considering the limitations of the current methods for reporting aggregate data accuracy, a weighted absolute percentage error (WAPE)-based method for calculating aggregate data reporting accuracy was found to resolve these issues. The WAPE weighs the sum of the absolute differences between the register and reported values by the register values:

$$\text{WAPE}=\;\frac{\sum_{i=1}^{n}\left|y_{i}-{\hat{y}}_{i}\right|}{\sum_{i=1}^{n}y_{i}}\times 100$$

where *n* represents the number of observations, $y_{i}$ represents the register value, and ${\hat{y}}_{i}$ represents the reported value.^5^ To prevent a zero-sum denominator when calculating the WAPE, one is added to both the register and reported values for a reporting period if the register value is zero. If the WAPE is greater than 100%, it is considered 100%. By using the register values as the weighting factor, the WAPE assigns greater importance to errors associated with high register values. It also equally penalizes overreporting and underreporting. Aggregate data reporting accuracy is calculated as 100%-WAPE, which we refer to as WAPE-based aggregate data reporting accuracy (WADRA). Like the PATH toolkit, the WADRA is categorized into one of three arbitrary reporting accuracy levels: low, medium, and high.

When the WADRA is applied to the three RDQA scenarios in Tables 2–4, the resulting aggregate reporting accuracies are distinct and accurately identify the health facility that has the greatest need for data quality improvement interventions. In the first scenario (Table 2), the health facility WADRA is 41% (low accuracy), whereas the aggregate accuracies of the other tools suggest high accuracy. The second scenario (Table 3) shows that the WADRA for Health Facility #1 (low caseload) is consistent with the high accuracy observed with the other tools; however, the WADRA for Health Facility #2 (high caseload) is 53% (low accuracy), contrasting with the outputs of the other tools, which indicate high accuracy. In the third scenario (Table 4), the PATH toolkit and WADRA are 0% (low accuracy) for Health Facility #3 and Health Facility #4, whereas the $\bar{VF}$ differ, suggesting low reporting accuracy (underreporting) at Health Facility #3 and high reporting accuracy at Health Facility #4, despite a difference of only one reported confirmed malaria case between the facilities. The WADRA in all three scenarios addressed the limitations of the current metrics and correctly identified the additional facilities that should be targeted for corrective actions and surveillance strengthening activities.

Aggregate data reporting accuracy, combined with other data quality indicators, is used at the district (or higher) level to develop and implement health facility-specific corrective action plans. At the national level, aggregate reporting accuracies help direct resources for data quality improvement activities to areas with low-accuracy facilities. Recently, WADRA was used to evaluate temporal trends in aggregate reporting accuracy using RDQA results in Zambia. A series of use cases were suggested, including guiding the selection and sampling of facilities for subsequent RDQAs; facilitating rapid reviews of RDQA performance, evaluating progress, and responding to issues; and providing insights into ways in which the RDQA process could be simplified to support the scale-up of coverage.^6^ Recognizing these potential use cases in the context of limited human and financial resources available for implementing RDQAs and data quality improvement activities, it is critical that the aggregate reporting accuracy metric correctly identifies the facilities with the greatest need for strengthening reporting practices. However, current aggregate reporting accuracy metrics are suboptimal in certain situations where, despite significant discrepancies between register and reported values, a health facility is considered to be reporting with high accuracy. This results in missed opportunities to initiate additional training, supportive supervision, or other tailored interventions. Here, the WADRA is introduced as a useful and straightforward metric for calculating aggregate data reporting accuracy using RDQA data, resolving the limitations associated with current approaches by using the register value as a weighting factor. Therefore, it is recommended that country malaria programs consider integrating the WADRA approach into their RDQA activities to guide decisions and target resources for improving data quality.
